# A Narrative Review of the Lesser Known Medications for Treatment of Restless Legs Syndrome and Pathogenetic Implications for Their Use

**DOI:** 10.5334/tohm.739

**Published:** 2023-03-02

**Authors:** Paul G. Yeh, Karen Spruyt, Lourdes M. DelRosso, Arthur S. Walters

**Affiliations:** 1 College of Health Professions, University of Texas Rio Grande Valley, Edinburg, Texas, US; 2 National Cancer Institute Cancer Control Research Training Program (T32/CA05771), University of Texas Health Science Center at Houston School of Public Health, Houston, Texas, US; 3 Université de Paris, NeuroDiderot INSERM, France; 4 Pulmonary and Sleep Medicine, University of California San Francisco-Fresno, Fresno, California, US; 5 Department of Neurology, Sleep Division, Vanderbilt University Medical Center, Nashville, Tennessee, US

**Keywords:** clonidine, dipyridamole, perampanel, amantadine, ketamine, carbamazepine, oxcarbazepine, lamotrigine, topiramate, valproic acid, levetiracetam, steroids, cannabis, bupropion

## Abstract

**Background:**

There are several well-known treatments for Restless Legs Syndrome (RLS), including dopamine agonists (pramipexole, ropinirole, rotigotine), anticonvulsants (gabapentin and its analogs, pregabalin), oral or intravenous iron, opioids and benzodiazepines. However, in clinical practice, treatment is sometimes limited due to incomplete response or side effects and it is necessary to be aware of other treatment options for RLS, which is the purpose of this review.

**Methods:**

We performed a narrative review detailing all of the lesser known pharmacological treatment literature on RLS. The review purposefully excludes well-established, well-known treatments for RLS which are widely accepted as treatments for RLS in evidence-based reviews. We also have emphasized the pathogenetic implications for RLS of the successful use of these lesser known agents.

**Results:**

Alternative pharmacological agents include clonidine which reduces adrenergic transmission, adenosinergic agents such as dipyridamole, glutamate AMPA receptor blocking agents such as perampanel, glutamate NMDA receptor blocking agents such as amantadine and ketamine, various anticonvulsants (carbamazepine/oxcarbazepine, lamotrigine, topiramate, valproic acid, levetiracetam), anti-inflammatory agents such as steroids, as well as cannabis. Bupropion is also a good choice for the treatment of co-existent depression in RLS because of its pro-dopaminergic properties.

**Discussion:**

Clinicians should first follow evidence-based review recommendations for the treatment of RLS but when the clinical response is either incomplete or side effects are intolerable other options can be considered. We neither recommend nor discourage the use of these options, but leave it up to the clinician to make their own choices based upon the benefit and side effect profiles of each medication.

## 1. Introduction

Restless Legs Syndrome (RLS) is a sensorimotor disorder that is most classically described as unpleasant sensations in the lower extremities and a resultant uncomfortable urge to move them, worsened by inactivity and nighttime, while relieved by movement [1]. Despite RLS being a rather common disorder in the general population, impacting between 5–15% of all adults [1], it is a misunderstood condition by patients and clinicians alike [2], and accordingly, it is often inadequately treated [2,3]. There are several well-known treatments for RLS including dopamine agonists (pramipexole, ropinirole, rotigotine), anticonvulsants (gabapentin and its analogues, pregabalin), oral or intravenous iron, opioids and benzodiazepines [1,2,3,4,5,6,7,8]. Some of these are FDA and European counterpart approved for the treatment of RLS.

However, in clinical practice, treatment is sometimes limited due to incomplete response or side effects; accordingly, it is necessary for clinicians and researchers to be aware of other lesser known treatment modalities for RLS. The purpose of this review, therefore, is to synthesize the existing evidence regarding alternative pharmacological treatment for RLS symptom management. Indeed, currently accepted first-line pharmacological treatment modalities for RLS, particularly dopamine agonists, have significant associated side effects and costs [9], which can preclude its use in many RLS patients [10]. First-line pharmacological dopamine agonists can instigate the undesired side-effect of augmentation where RLS symptoms are conversely exacerbated by the use of medications [10,11], tolerance over time, and loss of efficacy after 2–8 years of treatment [2,12,13]. The limitations of mainstream pharmacological treatment are highlighted by the fact that up to 65% of patients with RLS use complementary and alternative (CAM) treatment modalities, such as acupuncture, massage, and herbal remedies, for RLS symptomatic relief [14]. Moreover, none of the mainstream first-line treatment modalities are curative [2]. Therefore, we explored the lesser known pharmacological drugs for the management of RLS in this narrative review.

This is not an evidence-based review as by its very nature it is focusing on determining which alternative treatments exist for RLS patients. In these cases, the evidence for use does not approach the standard for wider endorsement, but does indicate some support from the literature for the use of these agents. Such treatments might be considered for isolated refractory cases when patients have failed multiple lines of standard treatment strategies. We do not aim to evaluate the quality of the publications cited as most systematic reviews do. The vast majority of the medications reviewed here do not have large sample sizes, double blinding, randomization, or other metrics of study rigor and quality. This article is meant, therefore, as a compilation reference for clinicians to consider alternative and adjunct treatment medications for refractory and/or severe cases of RLS. We have indicated which studies are open-label and which studies have double-blind comparison data. Where pathogenetic implications can be drawn from treatment studies, we have included that information as well. To our knowledge, this is the first scoping review of this kind on the treatment of RLS.

## 2. Methods

### 2.1. Eligibility Criteria

We reviewed all of the traditional Western treatment literature on RLS but did not include the more well-known treatments for RLS which have been extensively reviewed elsewhere and that are widely accepted as treatments for RLS in evidence-based reviews [1,2,3,4,5,6,7,8]. We included all non-standard pharmacological treatments from the literature for which there was some evidence for efficacy in RLS. Exclusions included dopamine agonists (pramipexole, ropinirole, rotigotine), gabapentin and its analogues (Pregabalin), oral or intravenous iron, low-dosage opioids (codeine, methadone, oxycodone, hydrocodone), and benzodiazepines (clonazepam) [1,2,3,4,5,6,7,8].

We also did not include non-pharmacologic, herbal preparations, vitamins, or minerals for the treatment of RLS which also have been reviewed elsewhere [15]. Furthermore, we did not review medical devices for RLS which employ compression, vibration, or electrical stimulation as these have been reviewed elsewhere as well [16].

#### 2.1.1. Search Methods

We searched the PubMed online database up to January 23^rd^, 2023 using the search term phrase, “Restless Legs Syndrome AND treatment.” After we found relevant articles, we then did another search where the name of the medication reported in the relevant articles was substituted in the phrase above as a way of capturing any relevant articles that may have been missed by the first search. We also supplemented our search with articles cited in the reference lists of articles picked up from our original literature search, when these secondary articles had not been picked up by the methodology above.

One major caveat to our search is that earlier studies may have employed objective evidence for therapeutic effects such as polysomnography and actigraphy or ad-hoc scales to rate RLS severity. Prior to 2003 studies did not have the advantage of employing a validated rating scale of symptoms such as the International Restless Legs Syndrome Study Group’s Symptom Rating Scale (the IRLS scale), a 10-question survey graded on a 4-point Likert scale where symptom severity score ranges from 1 (mild RLS) to 40 (very severe RLS) [2]. The IRLS currently serves as the major outcome for all academic and industry-sponsored studies on RLS [17]. Another caveat is that we have two studies that only looked at Periodic Limb Movement in Sleep (PLMS).

## 3. Pharmacological Therapeutic Agents for RLS

Up to 2023, we found 47 studies published fulfilling our inclusion criteria (Supplementary Figure S1 flowchart).

There were 9 single case reports. There were 11 case series. There was one survey study. There were 14 open-label studies without parallel or crossover groups. There were 8 double-blind cross-over studies of which 6 were randomized. There were 4 double-blind parallel studies of which 3 were randomized.

For three studies [18,19,20] we could not retrieve the full article but summarized the results from the abstract available on PubMed. We have indicated these articles by an asterisk in Table 1.

**Table 1:** Studies included.


NAME OF THE MEDICATION	AUTHOR	STUDY DESIGN	SAMPLE SIZE ENROLLED (TREATED)	DOSAGES	RESULTS	SIDE/ADVERSE EFFECTS REPORTED IN THE STUDY

**3.1. Reduction in Adrenergic transmission**						

Clonidine	Handwerker et al [21]	• case series	3	0.1 mg twice a day; 0.1 mg at bedtime; 0.3 mg at night	3/3 completely relieved	

	Bastani et al [23]	• case series	6	0.1 mg twice a day, and one needed this dose three times a day for optimal response	6/6 patients had moderate to marked improvement in their RLS symptoms	3/6 fatigue, drowsiness, dry mouth

	Bamford et al [24]	• open-label	7	initiated at 0.1 mg/night and increased to 0.2 mg/night the following week	provoking RLS	7/7 insomnia; 2/7 exacerbation RLS; 2/7 intolerable headaches, hypotension

	Ausserwinkler et al [19]*	• double-blind • parallel • placebo-controlled	20 (10 on drug and 10 on placebo)	0.075 mg bid twice daily	8/10 patients complete relief; 1 patient striking relief; 1 patient unchanged; 1 placebo subject mild alleviation	

	Wagner et al [25]	• double-blind • crossover • randomized • placebo-controlled	11 (5 on drug and 5 on placebo); 1 drop-out	a mean dose of 0.5 mg and maximum dose of 1.0 mg/day;	improved leg sensations, motor restlessness, daytime fatigue; quicker sleep onset; 7/10 patients more effect than placebo; 4/7 continued use after the study	decrease in REM sleep and sleep latency; increase in REM latency; 8/10 dry mouth, 6/10 decreased cognition, 5/10 sleepiness after dose, 4/10 constipation, 2/10 decreased libido and 2/10 headache

	Zoe et al [26]	• case report	1	0.9 mg/d	mild hypertension normalized	a dry mouth

	Prescriber guidelines: used cautiously in older individuals with accompanying cardiovascular disease [30]					

**3.2. Adenosine Transporter Inhibitor**						

Dipyridamole	Garcia-Borreguero et al [33]	• open-label • uncontrolled	15 (13 completed study)	began treatment with 100 mg dipyridamole (with up-titration to 400 mg if necessary) at 8:00 p.m.; mean effective dose: 281.8 ± 57.5 mg/day	6/13 full responders; 4/13 partial responders; improved PLMS index and sleep variables; improved per IRLS scale score, CGI and mSIT	abdominal cramps, diarrhea, dizziness, and flushing

	Garcia-Borreguero et al [34]	• double-blind • crossover • randomized • placebo-controlled	29 (14 on drug and 14 on placebo)	up-titration to a maximum of 300 mg at 9:00 PM [34]	improved per IRLS score, CGI, Medical Outcomes Study Sleep Scale and mSIT scores; Sleep variables and PLMS	mild side effects: abdominal distension, dizziness, diarrhea, and asthenia

	Prescriber guidelines: caution must be exercised in the administration of this agent, particularly in those who are receiving other antiplatelet therapy [35,36]					

**3.3. Glutamate**						

3.3.1 AMPA Receptor Antagonist						

Perampanel	Garcia-Borreguero et al [38]	• open-label • uncontrolled	22 (20 completed study)	2 mg- 4 mg at 19:00; mean dose: 3.8 ± 1.2 mg/day	improved IRLS scale score, mSIT, PLMS index; 12/20 full responders; 4/20 partial responders	6/20 dizziness, 2/20 somnolence, 1/20 headache and 5/20 irritability; 1 patient discontinued because of irritability; 1 patient discontinued because of lack of efficacy

	Prescriber guidelines: serious psychiatric side effects are common [39]					

3.3.2. NMDA Receptor Antagonists						

Amantadine	Evidente et al [40]	• open-label • uncontrolled	21	100 mg- 300 mg/day; mean dose: 227 mg/day	11/21 mild subjective improvement; 6/21 strong subjective improvement RLS rating scale	3/21 drowsiness, 2/21 fatigue, 2/21 insomnia, 1/21 dry mouth, 1/21 leg edema and 1/21 weight loss; 1 patient discontinued because of leg edema; 1 patient discontinued because of fatigue, drowsiness and weight loss

	Prescriber guidelines: possibility of daytime sleep attacks, impulse control disorders and hallucinations [41]					

Ketamine	Kapur et al [42]	• case series	2	30–40 mg bid mixed with 50 ml water twice a day given orally	significant improvement in sleep and RLS symptoms	

	Prescriber guidelines: possibility of cardiac arrhythmia, hypotension, dependence, respiratory depression, and hallucination[43]					

**3.4 Anticonvulsants Other Than Gabapentin And Pregabalin**						

Carbamazepine	Lundvall et al [44]	• double-blind • crossover • randomized • placebo-controlled	6 (with 2 only for three weeks on placebo)	200 mg; 1 tablet in the morning and 2 tablets in the evening	3/6 were subjective responders; drop of 21% severity score compared placebo value; 3 patients continued after study	1 patient experienced mild gastritis; 1 subject experienced sweating, dry mouth and vomiting

	Telstad et al [45]	• double-blind • parallel • randomized • placebo-controlled	174 (84 on drug and 90 on placebo)	100 mg tablets were up-titrated to a maximum dosage of 300 mg at bedtime; median dose: 236 mg	the frequency of RLS attacks/week decreased	34/84 subjects on carbamazepine including 6 withdrawals; 20/88 subjects on placebo including 2 withdrawals

Oxcarbazepine	Oztürk et al [46]	• case report	1	150 mg bid twice a day	improvement per IRLS scale score	side effects were not reported in this case report

	Jimenez-Trevino et al [47]	• case series	3	300 mg up-titration to 600 mg at bedtime	excellent remission	1 patient experienced mild dizziness but during the first week of treatment only

	Prescriber guidelines: boxed warning for carbamazepine and oxcarbazepine for the development of Stevens-Johnson syndrome and for the development of aplastic anemia and agranulocytosis; hyponatremia [48,49]					

Lamotrigine	Youssef et al [50]	• open-label • uncontrolled	4 (3 completed the study)	250 mg –500 mg/day; mean dose: 360 mg/day	2/3 patients reported a sustained decrease in sensations, leg kicking and restlessness; 3/3 patients continued	2/3 pruritis, 1/3 dizziness and 1/3 chest pain; 1 withdrawal due to dizziness

	McMeekin et al [51]	• case series	7	slowly up-titrated	successful treatment	

	Prescriber guidelines: caution in patients with heart disease; boxed warning regarding the development of Stevens -Johnson Syndrome and other severe skin reactions [52]					

Topiramate	Pérez Bravo et al [20]*	• open label • uncontrolled	19	a mean effective dosage of 42.1 mg	improvement 19 patients	weight loss; hepatic or renal impairment

	Romigi et al [53]	• case series	2	slowly titrated up to 50 mg bid; up to 100 mg bid	provoking RLS	provoking RLS

	Bermejo et al [54]	• case series	2	titrated up to 50 mg bid; titrated up to 75 mg/day	provoking RLS	provoking RLS

	Prescriber guidelines: a warning for use in patients with hepatic or renal impairment; possibility of attention, memory and language problems [55]					

Valproic Acid	Ehrenberg et al [56]	• open-label (for PLMS and not RLS) • uncontrolled	6	125–600 mg at bedtime	subjective improvement in daytime alertness and sleep (REM sleep unchanged)	1/6 short-term side effects; 1/6 weight gain

	Eisensehr et al [57]	• double-blind • crossover (three comparator groups- Valproic Acid, L-DOPA and placebo) • randomized • placebo-controlled	20	600 mg slow-release valproic acid or 200 mg slow-release levodopa + 50 mg benserazide 90 minutes before bedtime	decrease of intensity and duration of RLS symptoms was more pronounced with valproic acid than levodopa	9/20; levodopa increased arousals not associated with PLMS; pressure in the chest, flatulence, difficulty falling asleep, drowsiness, edema, finger pain, headache, blurred vision, hand tremor and hair loss

	Prescriber guidelines: boxed warning for the possibility of hepatotoxicity, pancreatitis, and congenital malformations [58]					

Levetiracetam	Della Marca et al [59]	• case series	2	1000 mg at bedtime and 500 mg at bedtime	2 patients improved per IRLS scale score, SIT, ESS, and in the sleep latency, sleep efficiency and PLMS index; both continued on the medication	

	Gagliano et al [60]	• open-label • uncontrolled	7	titrated up to 50–60 mg/kg/day in two separate doses (in children)	improvement per IRLS scale score, sleep quality and fewer awakenings and restorative sleep; EEG focal interictal epileptic discharges in sleep	mild: 2/7 increase in appetite and in daytime irritability

	Prescriber guidelines: possibility of the development of rare but severe cutaneous reactions such as the Stevens-Johnson syndrome; renal failure; psychiatric side effects, including psychosis, paranoid ideation, and behavioral instability, may also occur [61]					

**3.5. Steroids And The Auto-Immune, Anti-Inflammatory Link**						

Hydrocortisone	Hornyak et al [63]	• double-blind • crossover • randomized • placebo-controlled	10 (5 on drug and 5 on placebo)	40 mg prior to a one hour SIT	decrease in sensory leg discomfort; 5/10 subjective improvement	none

Oral Prednisolone	Oscroft et al [64]	• case report	1	15 mg/day; tapered to a minimum effective dose of 8 mg/day	Improvement per IRLS scale score; ESS	

	Prescriber guidelines: potential long term consequences such as osteoporotic fractures, hypertension, steroid-induced diabetes and changes in physical appearance such as moon face and buffalo hump [64,65,66]					

**3.6. Other Medications And Substances**						

Cannabinoids	Megelin et al [67]	• case series	6	occasional and recreational smoking; sublingual administration of cannabidiol	6 patients well tolerated	1/6 nausea therefore restricted smoking to periods with symptom severity exacerbation

	Samaha et al [68]	• survey	192 (15 responded to use cannabis	cannabis use	9/15 reported improvement	

	Prescriber guidelines: monitoring for potential CNS depression, hepatic impairment, and suicidal ideation [69]					

Bupropion	Park et al [71]	• case report	1	150 mg daily up to 300 mg	improvement per IRLS scale score; total resolution	

	Lee et al [72]	• case report	1	150 mg daily	“feeling good”; able to initiate and maintain sleep for 7–8 hours nightly	

	Kim et al [73]	• case series	3	150 mg sustained-release (SR) bupropion morning once per day	2/3 Improvement per IRLS scale score, sleep (un)changed minimally	

	Bayard et al [74]	• double-blind • parallel • randomized • placebo-controlled	60 (29 on drug and 31 on placebo); with drop-outs at 3 and 6 weeks	150 mg XL/day two hours before bedtime	improvement per IRLS scale score only at 3 weeks	withdrawals: 1/29 - nausea; 1/29 – miscarriage; 2/29 – no reason; 2/31 – nausea; 1/31 irritable mood; 1/31 – no reason

	Vishwakarma et al [75]	• double-blind • parallel (three active comparators Bupropion, ropinirole and iron) • randomized	103 (30 in each group); 13 drop-outs	300 mg/day, ropinirole 0.5 mg/day or oral iron 150 mg elemental formulation along with vitamin C	all groups improvement per IRLS scale score (particularly the ropinirole group), RLS Quality of Life	9/99 patients; nausea, dizziness, tremor, tingling sensation in palm and sole, gastritis, constipation, and weight gain

	Prescriber guidelines: a boxed warning alerting prescribers to the possibility of suicidal thoughts and behavior [76]					

Baclofen	Guilleminault et al [77]	• double-blind (for PLMS only – not RLS) • placebo at baseline was used as comparator to medication given thereafter • crossover • placebo-controlled	5	20 mg up-titration to 160 mg at 9:45 PM; dosages of 20 mg and 40 mg were the most efficacious	Improvement in 5 patients; effect on sleep was dose related: as dosages increased, delta sleep progressively increased and REM sleep decreased	2/5 nausea, during the night at the 80 mg dosage, which may explain the overall decrease in total sleep time (TST) at this dosage, and the moderate increase in wake after sleep onset

	Sandyk et al [78]	• case report	1	10 mg nightly	Patient improved sleep, daytime somnolence, RLS	

	Brown et al [79]	• case report	1	1204 mcg/day intrathecal baclofen		no improvement

	Prescriber guidelines: very slowly withdrawing patients on intrathecal baclofen due to the possibility of developing high fever, confusion and, in some cases, rhabdomyolysis, multiple organ failure and death [80]					

Physostigmine	Alpert et al [81]	• case report	1	1 mg IV	Sixty to 90 s after the administration all leg movement ceased	

	Peacock et al [82]	• case report	1	1 mg IV	3 minutes after the administration all symptoms attenuated	

	Prescriber guidelines: contraindications of using physostigmine in patients with gastrointestinal or genitourinary obstruction, asthma, cardiovascular disease, and may cause arrythmias, or seizures [83]					

Orphenadrine Citrate	Popkin et al [18]*	• open-label • uncontrolled	32; (20 on drug) 12 drop-outs	oral dosage of 100 mg (occasionally 200 mg) daily for 1 or 2 divided doses in the evening	16/20 long-term excellent results (subjectively)	12/32 discontinued due to gastrointestinal intolerance

	Prescriber guidelines: not to be used in older adults because of an increased risk of anticholinergic effects, sedation and risk of fracture; caution in patients with heart disease and drug or alcohol abuse [84]					

Rifaximin	Weinstock et al [86]	• open-label • uncontrolled	13	400mg three times daily; 1200 mg/day for 10 days	6/13 patients complete resolution; 86% overall long term improvement	

	Weinstock et al [87]	• open-label • uncontrolled	14	1200 mg/day for 10 days followed by 400 mg every other day	global improvement in 9/14 patients per IRLS score	

	Prescriber guidelines: warnings to observe for allergic reactions and super-infections with other organisms [88]					

Botulinum toxin (BTX)	Rotenberg et al [89]	• case series	3	BTX-A (100 units per cc) was injected into each of his tibialis anterior muscles bilaterally, in divided doses of 25 units, with a total of 50 units per muscle; a total of 320 units of BTXA (100 units per cc) bilaterally in his lumbar paraspinal muscles (40 units per site, in 2 sites per side), his gastrocnemii (20 units per site and 2 sites per leg), and his quadratus femorii (20 units per site and 2 sites per leg); a total of 70 units of BTX-A (50 units per cc) were administered subcutaneously in the dysesthetic areas (5 units per site and 7 sites per leg)	Improvement subjective and ESS	1/3 patients experienced residual weakness

	Mittal et al [90]	• double-blind • crossover • randomized • placebo-controlled	24 (8 on drug and 13 on placebo); 3 drop-outs	100 units of Incobotulinumtoxin A (IncoA)	21 patients improved per IRLS, ESS and the sleep questionnaire score; 7/21 definite or marked improvement on CGI	none with residual weakness

	Agarwal et al [91]	• open-label • uncontrolled	8	received 50 units of onabotulinum toxin A, two points of injection were used per tibialis anterior	8 cases; improvement per PGI-S	none with residual weakness

	Ghorayeb et al [92]	• open-label • uncontrolled	27 (26 completed the study)	20 intradermal injections of 0.05 ml of BoNT/A	6/27 improved per IRLS scale score; 10/27 improvement per CGI-I scores	7/27 patients experienced transient limb weakness; 1/27 reported to have diplopia; 1 withdrawal due to lack of efficacy after week 12

	Nahab et al [93]	• double-blind • crossover • placebo-controlled	6	IM injections of 70–320 mouse units (mU) of botulinum toxin type A	6/6 patients without improvement per IRLS scale or CGI	2/6 patients experienced weakness

	Prescriber guidelines: black box warning of dysphagia and breathing difficulties, bacteriuria, urinary retention and tract infection, and anaphylaxis hypersensitivity reaction [95]					

Selegiline	Grewal et al [96]	• open-label(for PLMS only – not RLS) • retrospective • uncontrolled	31	5mg bid for 2 weeks (n = 5 at end of follow-up period); increased to 10mg bid for next 2 weeks (n = 5 at end of follow-up period); then lastly increased to 15mg bid for last two weeks (n = 21 at end of follow-up)	PLMS were suppressed in the total group by 21.2/hr (pretreatment 35.6/hr and post-treatment 14.5/hr) (p < 0.0005) as determined by polysomnography The subgroup of 21 patients who were on 15mg bid treatment dosage saw PLMS reduction by 20.7/hr (p < 0.0005)	4/31 patients experienced slight feelings of disconnection, nervousness, and nausea

	Prescriber guidelines: using selegiline include orthostatic hypotension, vertigo, palpitation, suicidal thinking/behavior, dyskinesia, psychosis, and CNS depression [97]					


*: Information based on abstract; **CGI:** Clinical Global Impression; **ESS:** Epworth Sleepiness scale; **IRLS scale:** International Restless Legs Syndrome Study Group’s Symptom Rating Scale; **IV:** intravenous; **mSIT:** Multiple Suggested Immobilization Tests; **PGI-S:** Patient Global Impression-Severity; **PLMS:** Periodic Limb Movements in Sleep; **RCT:** Randomized Controlled Trial; **SIT:** Suggested Immobilization Test. Side/Adverse Effects from the studies themselves are listed vertically in the column Side/Adverse Effects. Side effects from the broader literature are listed horizontally under each group of medications.

**Figure 1:**
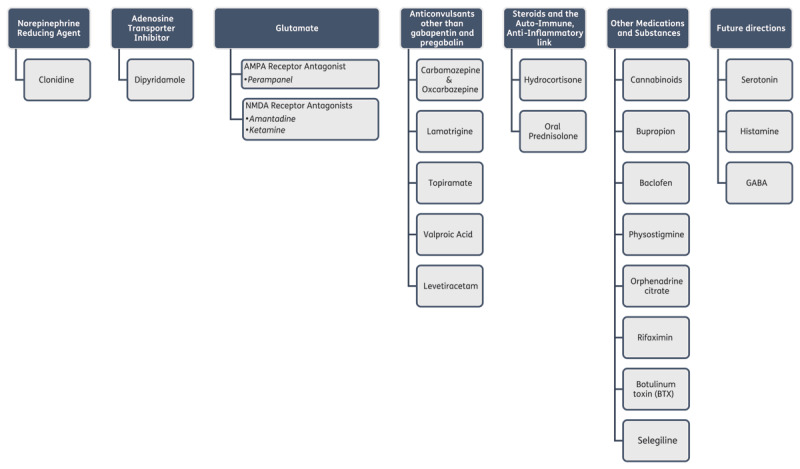
Scheme of reviewed drugs.

The results are summarized in the text and the table. In the Table 1 side effects from each particular study are indicated in the column “Side/Adverse Effects” whereas side effects and adverse events from the literature are indicated in the horizontal row beneath each particular medication. In Table 2 studies applying the IRLS score are summarized. A schematic representation of the reviewed drugs is given in Figure 1.

**Table 2:** Studies employing the International Restless Legs Scale (IRLS) (mean ± standard deviation): Baseline and endpoint scores are provided.


DRUG	AUTHOR	METHOD	IRLS SCALE SCORE	CGI SCORE	MSIT

Dipyridamole	Garcia-Borreguero et al [33	baseline	23.4 ± 4.6	3.5 ± 0.5	26.6 ± 9.8 (3 tests)

		endpoint	10.7 ± 4.5	1.7 ± 0.7	18.9 ± 7.5 (3 tests)

Dipyridamole	Garcia-Borreguero et al[34	baseline	24.1 ± 3.1	3.2 ± 0.9	15.6 ± 8.6 (subjective scale)

		endpoint	11.1 ± 2.3	1.3 ± 0.6	28.3 ± 7.2 (subjective scale)

Perampanel	Garcia-Borreguero et al[38	baseline	23.7 ± 4.2	3.6 ± 0.48	44.2±14.5 (4 tests)

		endpoint	11.5 ± 5.2	1.8 ± 0.74	27.3±10.7 (4 tests)

Oxcarbazepine	Oztürk et al[46	case 1 baseline	32		

		case 1 endpoint	19		

Levetiracetam	Della Marca et al[59	case 1 & 2 baseline	34 & 27		39 & 45 SIT PLMS index (events/h)

		case 1 & 2 endpoint	22 & 18		11 & 21 SIT PLMS index (events/h)

Levetiracetam	Gagliano et al[59	baseline	18.9 ± 2.6		

		endpoint	4.7 ± 4.9		

oral prednisolone	Oscroft et al[64	case 1 baseline	22		

		case 1 endpoint	14		

Bupropion	Park et al[71	case 1 baseline	26		

		case 1 endpoint	14		

Bupropion	Kim et al[73	case 1 & 2 & 3 baseline	24 & 22 & 23		

		case 1 & 2 & 3 endpoint	9 & 10 & 0		

Bupropion	Bayard et al[74	baseline	26.3 ± 5.4		

		endpoint	15.9 ± 9.1		

Botulinum toxin (BTX)	Mittal et al[90	endpoint	27 ± 7.13		

Botulinum toxin (BTX)	Ghorayeb et al[92	baseline	31.1 ± 4.7		

		endpoint	22.3 ± 8.5		

		follow-up	26.1 ± 7.2		

Botulinum toxin (BTX)	Nahab et al[93	baseline	27 ± 4.8	4.3 ± 0.8	

		improvement	5 ± 6	3.7 ± 1.4	


**CGI:** Clinical Global Impression; **IRLS scale:** International Restless Legs Syndrome Study Group’s Symptom Rating Scale; **mSIT:** Multiple Suggested Immobilization Tests For all studies that employed the IRLS data could be extracted, except for Vishwakarma et al [75], Mittal et al [90 and Evidente et al [40].

We used a variable way of grouping of the medications that we review. In some cases medications are grouped by the neurotransmitter that they affect, particularly when these neurotransmitters play a potential pathogenetic role in RLS. In other cases they are grouped by their general therapeutic effect, e.g., anticonvulsants, when the mechanism of action does not involve a neurotransmitter.

### 3.1. Reduction in Adrenergic Transmission

#### 3.1.1. Clonidine

Most of the experimental literature on clonidine for use in RLS was accrued in the late 1980’s up to the mid 1990’s [19,21,22,23,24,25,26]. Studies are small and three of the initial open-label studies/case reports reported benefit [21,22,23] with a 4^th^ study reporting no benefit [24]. There are only two double-blind studies of clonidine in RLS of which we are aware, both of which reported benefits [19,25]. Clonidine has also been used in childhood RLS with accompanying ADHD, given the independent beneficial effect of clonidine on ADHD [1,2,3,4,5,6,7,8,27]. Clonidine was originally designed to control hypertension through its general overall reduction in adrenergic response [28]. The positive response of RLS to clonidine in conjunction with the well-known observation that selective noradrenergic reuptake inhibiting antidepressants (SNRIs) make RLS worse [29] are compatible with the hypothesis that hyperadrenergicity is pathogenic to RLS.

The first double-blind study of clonidine for RLS was in patients with chronic kidney insufficiency [19]. Clonidine 0.075 mg bid was administered to 10 patients while 10 received a placebo [19]. In the treatment group, 8 out of 10 patients experienced complete relief of symptoms and another patient experienced striking relief [19]. In the placebo group, only one subject experienced a mild alleviation of symptoms [19]. The second double-blind study of clonidine for RLS was a crossover study of 10 patients with idiopathic RLS at a mean dose of 0.5 mg and a maximum dose of 1.0 mg/day [25]. Subjective reports of symptoms as well as polysomnographic and actigraphic information were collected. By the patient report, leg sensations (p = 0.02) and motor restlessness (p = 0.001) were improved on clonidine compared to the placebo. By polysomnography, sleep onset was quicker with clonidine (p = 0.006) [25]. With clonidine, there was also a non-statistical trend toward an increase in stage 3 & 4 sleep by polysomnography (p = 0.067) and a trend toward a decrease in motor activity as measured by actigraphic recordings (p = 0.1) [25]. Globally, 7 out of 10 patients felt clonidine was more effective than placebo [25]. Four patients chose to continue clonidine after the study [25]. Some negative impacts on sleep were also noted with a decrease in REM sleep and an increase in REM latency on polysomnography [25]. In some cases, higher dosages of clonidine up to 0.9 mg/day have been reported to be necessary and tolerated [26]. In the double-blind study a large number of patients had side effects from clonidine but these were considered mild and decreased with dosage reduction [25]. Side effects included dry mouth, decreased cognition, sleepiness after dose, constipation, decreased libido and headache [25]. No patient discontinued the study due to side effects [25]. Because of its antihypertensive effect, clonidine must be used cautiously in older individuals with accompanying cardiovascular disease [30].

### 3.2. Adenosine Transporter Inhibitor

#### 3.2.1.Dipyridamole

Brain iron deficiency is thought to be almost universal in human RLS. The interest in the therapeutic use of dipyridamole developed in concert with basic science animal experiments, which suggested that RLS in the presence of brain iron deficiency is characterized by a hypoadenosinergic state [31,32]. Dipyridamole increases extracellular adenosine by inhibiting adenosine transporters [31,32] and was initially found to be effective in RLS first in an open-label trial [33] and subsequently in a double-blind crossover trial of 28 patients with up-titration to a maximum of 300 mg of dipyridamole [34]. In the double-blind trial, there was an improvement in the IRLS scale (–13 points versus –5 points with placebo; p < 0.001) [34]. Clinical Global Impression scale (CGI) (–1.9 points versus –0.4 points; p < 0.001), Medical Outcomes Study Sleep Scale (p < 0.001), and Multiple Suggested Immobilization Test scores (mSIT) all improved as well (+16 points versus +28 points, p < 0.001) [34]. Sleep variables (sleep latency of 20 minutes versus 28 minutes; p = 0.007 and N3 stage sleep at 15% versus 9% of total sleep time; p < 0.001) and PLMS index (7.5 vs. 19.1 PLM; p < 0.001) also improved on polysomnography [34]. Side effects in the double-blind study were mild and included abdominal distension, dizziness, diarrhea, and asthenia. None of the side effects led to discontinuation from the study [34]. As dipyridamole was originally designed as an antiplatelet agent for the treatment of ischemic stroke, caution must be exercised in the administration of this agent, particularly in those who are receiving other antiplatelet therapy [35,36].

### 3.3. Glutamate

As aforementioned, based on animal experiments, brain iron deficiency in RLS is thought to cause a hypoadenosinergic state [31,32]. In the presence of iron deficiency, this is particularly reflected in the downregulation of the Adenosine A1 receptors which are responsible for the inhibition of the release of glutamate. Thus low adenosine results in the release of more glutamate from cortico-striatal neurons at the level of the striatum and a hyperglutaminergic state [31,32]. This is also evidenced in imaging studies of human RLS where glutamate is shown to be increased in thalamus [37]. Thus, it would seem that glutamate antagonists might play a therapeutic role in RLS. There are two glutamate receptor subtypes – NMDA (N-methyl-D-Aspartate) and AMPA (alpha-amino-3-hydroxy-5-methyl-4-isoxazolepropionic acid). The data below illustrates evidence that the blockade of each of these two types of glutamate receptors results in the improvement of RLS [38,39,40,41,42].

#### 3.3.1. AMPA Receptor Antagonist

##### 3.3.1.1 Perampanel

Perampanel is an anticonvulsant the major effect of which is to block AMPA receptors [38]. In a prospective open-label two-month trial, 20 patients with idiopathic RLS were treated with 2 mg- 4 mg of perampanel [38]. At the end of 8 weeks, the IRLS scale score had dropped from 23.7 to 11.5 (p < 0.001), twelve of the patients had a complete response defined as a 50% drop in IRLS scale score and 4 responded partially (p < 0.001) [38]. On polysomnography, the mean PLMS index dropped from 27.8 to 4.36 (p < 0.001) [38]. A double-blind placebo-controlled trial of perampanel is in the planning stage [38]. In the study side effects included dizziness, somnolence, headache, and irritability [38]. One patient had to discontinue the study because of irritability and one had to discontinue because of a lack of efficacy [38]. Serious psychiatric side effects of perampanel are common and patients should be monitored carefully for their development [39].

#### 3.3.2. NMDA Receptor Antagonists

##### 3.3.2.1.Amantadine

Amantadine is an NMDA receptor antagonist and was developed first as an antiviral agent and secondarily as an adjunctive treatment for Parkinson’s Disease because of its pro-dopaminergic properties [40]. In an open-label trial, 21 patients with RLS were treated with 100 mg- 300 mg/day [40]. Patients were rated pre- and post-treatment on a 0 –10 RLS scale [40]. Patients also rated their degree of response on a continuous scale from 0% (no improvement) to 100% (complete improvement) [40]. Eleven of the 21 patients had subjective improvement with amantadine with the degree of response ranging from 20%–100% (mean 69%) among responders [40]. Six had a 95%–100% improvement [40]. The RLS scale score for the entire group of 21 patients dropped from a mean of 9.8 to 6.6 post-treatment (p = 0.001) [40]. The duration of response was a mean of 3.6 months at the time of the publication of the study with 9 responders still on the medication [40]. In the study, side effects included drowsiness, fatigue, insomnia, dry mouth, leg edema, and weight loss. One patient discontinued the study because of leg edema and one patient discontinued the study because of fatigue, drowsiness, and weight loss [40]. Because amantadine also has dopamine agonist properties, prescribers should be aware of the possibility of daytime sleep attacks, impulse control disorders, and hallucinations [41].

##### 3.3.2.2. Ketamine

Ketamine is another NMDA receptor antagonist which is used as an adjunct to anesthesia induction [42]. In a case report, two patients with RLS were treated with ketamine 30–40 mg bid mixed with 50 ml water given orally [42]. Both patients noted an immediate improvement in RLS symptoms as documented on a visual analogue scale after administration of ketamine (improvement from 6/10 to 2/10 and from 7/10 to 2/10, respectively), and both patients were continuing on ketamine for 1–6 months at the time of publication (no p-value provided) [42]. Neither of the subjects in this study experienced side effects [42]. This report comes from the anesthesia literature and, given the unusual oral formulation for its administration in this study, it is recommended that the medication be given in consultation with an anesthesiologist [42,43]. Other side-effects to be aware of for prescribers include the possibility of laryngospasm, cardiac arrhythmia, hypotension, dependence, respiratory depression, and hallucination [43].

### 3.4. Anticonvulsants Other Than Gabapentin And Pregabalin

A variety of anticonvulsants with a variety of different mechanisms have been found to be useful in RLS management.

#### 3.4.1. Carbamazepine & Oxcarbazepine

Carbamazepine and oxcarbazepine are the two medications that are the best studied of the anticonvulsants covered in this review in regard to RLS [44,45,46,47]. Oxcarbazepine is a structural analog of carbamazepine and both are blockers of voltage-gated sodium channels. Oxcarbazepine has less potential for drug interactions than carbamazepine and thus has a good tolerability profile [46]. In a double-blind crossover trial of carbamazepine 200 mg 1 tablet in the morning and 2 tablets in the evening or a matching placebo were given in two 4-week phases for six patients with RLS [44]. The number of attacks of RLS symptoms was recorded on a daily basis as was their severity on a 0–3 scale [44]. Three of the patients were classified as subjective responders and, in the responders, the severity of the RLS symptoms during carbamazepine treatment was only 21% of the corresponding value of placebo [44]. However, even in subjective non-responders, the mean severity of RLS during carbamazepine was lower than with placebo and all 6 patients treated with carbamazepine had lower mean severity scores during carbamazepine treatment compared to placebo (11.2 severity score vs. 14.1 with placebo; no p-value reported) [44]. No patient felt better on a placebo than on carbamazepine. Three patients decided to continue treatment after the trial [44]. In this study, one patient experienced mild gastritis, and another subject experienced seating, dry mouth, and vomiting [44].

In the second double-blind trial of carbamazepine in RLS, 174 patients were entered in a parallel design into the study of which 84 received carbamazepine and 90 received a placebo [45]. Carbamazepine 100 mg tablets were up-titrated to a maximum dosage of 300 mg [45]. The number of attacks of RLS during each week of the study was recorded over the 5 weeks of the trial [45]. The severity of the symptoms to the degree that they interfered with sleep was recorded on a visual analogue scale [45]. Another visual analogue scale recorded the subjects’ subjective evaluation of their response [45]. Maximal improvement occurred during the third through fifth weeks of the trial with the frequency of RLS attacks/week being less for a drug than placebo at 3 weeks (p = 0.04) and at 5 weeks (p = 0.03) [45]. The severity of RLS symptoms as measured on the visual analogue scale for sleep disturbance from RLS paralleled these results, being less for a drug than placebo at 3 weeks (p < 0.01) and 5 weeks (p < 0.01) [45]. More patients on carbamazepine regarded their response as being good (p < 0.01) or excellent (p < 0.01) compared to placebo on the subjective visual analogue scale [45]. In the larger study of carbamazepine for RLS, 34/84 subjects on carbamazepine experienced side effects including 6 withdrawals, and 20/88 subjects on placebo also experienced side effects including 2 withdrawals. According to the authors, none of the side effects were serious [45].

In a case report, oxcarbazepine 150 mg bid was found to cause remission in RLS symptoms in a 36-year-old male where the RLS was thought to be induced by paroxetine taken for the obsessive-compulsive disorder [46]. In this particular case, the psychiatric symptoms were severe, and the paroxetine dosage could not be lowered enough to effectively decrease RLS symptoms without the addition of oxcarbazepine [46]. Side effects were not reported in this case report [46]. In three case reports, dosages of 600 mg, 300 mg, and 600 mg, respectively, at bedtime resulted in excellent remission of RLS symptoms, which was continuing after 6 months of therapy at the time of publication (no scale or p-value reported) [47]. One of the 3 patients in this series had mild dizziness but during the first week of treatment only. No other side effects were reported [47]. Treatment guidelines include a boxed warning for carbamazepine for the development of Stevens-Johnson syndrome and for the development of aplastic anemia and agranulocytosis. These same side effects have occurred with oxcarbazepine. Hyponatremia can also occur with either medication [48,49].

#### 3.4.2. Lamotrigine

There are 2 reports in the literature on the successful use of lamotrigine in the treatment of RLS [50,51]. In the first of these reports, 3 patients completed the study [50]. There were 3 phases of the study which were a 3-day drug-free baseline phase, a 3-month phase to titrate lamotrigine, and a 1-month phase on a stable dose [50]. Patients recorded evaluations of RLS symptoms and sleep on a daily basis [50]. Three-day objective assessments were done at the end of each phase and these included continuous monitoring with activity meters and a modified one-hour SIT each evening [50]. Final dosages ranged from 250 mg–500 mg/day [50]. Two patients reported a sustained decrease in sensations, leg kicking, and restlessness (no p-value provided), and all 3 patients felt that lamotrigine benefited them and continued using it after the study [50]. Although there was a trend toward improvement in the level of activity and periodic limb movement while awake (PLMW) as measured by actigraphy and the mSIT, the results did not reach statistical significance (no scores or p-value provided), perhaps due to the small sample size and the known variability in these measures [50]. In this study side effects included pruritis, dizziness, and chest pain and there was one withdrawal from the study due to dizziness [50]. In the second study where lamotrigine was used in the treatment of RLS, 7 patients with bipolar disorder demonstrated a broad spectrum of symptoms including RLS, parkinsonism, bruxism, jaw clenching, and night sweats. The authors report successful treatment of all these symptoms with lamotrigine (no scores or p-value provided) [51]. Treatment guidelines include a boxed warning regarding the development of Stevens-Johnson Syndrome and other severe skin reactions. Lamotrigine should be up-titrated slowly to avoid such complications [51,52]. Lamotrigine should also be used in caution in patients with heart disease [52].

#### 3.4.3. Topiramate

Nineteen patients in an open-label study of topiramate showed improvement in RLS symptoms (at 90 days, 63.2% of patients had reduced their severity of RLS from moderate to mild) at a mean dosage of 42.1 mg (p < 0.05) [20]. On the other hand, two separate case series reports written by different authors showed that topiramate induced the symptoms of RLS in 2 cases each for a total of 4 cases of topiramate-induced RLS [53,54]. Weight loss stands out as an important side effect of topiramate and did so in the study of 19 patients [20]. Treatment guidelines indicate a warning for use in patients with hepatic or renal impairment. Topiramate also has been associated with attention, memory, and language problems [55].

#### 3.4.4. Valproic Acid

Valproic acid was originally investigated as a treatment for PLMS [56] and subsequently for RLS [57]. Twenty patients with idiopathic RLS were treated with 600 mg slow-release valproic acid or 200 mg slow-release levodopa + 50 mg benserazide in a randomized double-blind crossover trial [57]. The decrease in intensity and duration of RLS symptoms was more pronounced with valproic acid (RLS intensity score of 3.8 vs. 5.5 with placebo; p = 0.022) than with levodopa (RLS intensity score of 4.4 vs. 5.5 with placebo; p > 0.05) [57]. In addition, levodopa, but not valproic acid, significantly increased arousals not associated with PLMS (levodopa arousal index of 15.1 vs. 9.4 with placebo; p =0.002) [57]. Side effects in this study included pressure in the chest, flatulence, difficulty falling asleep, drowsiness, edema, finger pain, headache, blurred vision, hand tremor, and hair loss [57]. Treatment guidelines included a boxed warning for the possibility of hepatotoxicity, pancreatitis, and congenital malformations [58].

#### 3.4.5. Levetiracetam

In a case-report study of two patients who had experienced augmentation with pramipexole after an initial improvement, treatment with levetiracetam brought patients back to the level of improvement they had experienced in RLS symptoms with pramipexole prior to augmentation (no p-value provided) [59]. This improvement was reflected in the IRLS scale score (21 with Pramipexole, 35 with augmentation, and then 22 with Levetiracetam in patient 1; 15 with Pramipexole, 31 with augmentation, and then 18 with Levetiracetam in patient 2). This trend was repeated for the number of PLMW in the Suggested Immobilization Test (SIT) (Patient 1: 26 events/hour with Pramipexole pre-augmentation, 11 with Levetiracetam post-augmentation; Patient 2: 29 events/hour with Pramipexole pre-augmentation, 21 with Levetiracetam post-augmentation), in the Epworth Sleepiness scale (ESS) (Patient 1: 10 with Pramipexole pre-augmentation, 10 with Levetiracetam post-augmentation; Patient 2: 9 with Pramipexole pre-augmentation, 11 with Levetiracetam post-augmentation), and the sleep latency (Patient 1: 34 minutes with Pramipexole pre-augmentation, 27 minutes with Levetiracetam post-augmentation; Patient 2: 9 minutes with Pramipexole pre-augmentation, 11 minutes with Levetiracetam post-augmentation), and PLMS index on polysomnography (Patient 1: 23.1 events/hour with Pramipexole pre-augmentation, 10.9 with Levetiracetam post-augmentation; Patient 2: 31.5 events/hour with Pramipexole pre-augmentation, 12.6 with Levetiracetam post-augmentation) (no p-values provided) [59]. The dosages of levetiracetam were 1000 mg at bedtime and 500 mg at bedtime for the two patients [59]. At the time of publication, the subjects were continuing on levetiracetam for 26 and 21 months respectively with continuing satisfactory control of their RLS symptoms [59]. Side effects were not reported in the two patients but levetiracetam was said to be well tolerated by the authors [59]. In a case report of 7 children all of whom had RLS and ADHD and focal interictal epileptic discharges (IEDs) on EEG in sleep, levetiracetam was titrated up to 50–60 mg/kg/day in two separate doses [60]. At a 3 and 6-month follow-up, all children showed significant improvement in RLS severity as reflected in the IRLS scale score (baseline mean score 18.9 vs. 3-month mean score of 6.4; p =0.01; baseline score of 18.9 vs. 6-month mean score of 4.7; p =0.01) [60]. The parents reported improved sleep quality and fewer awakenings and restorative sleep in their children [60]. EEG focal interictal epileptic discharges in sleep also improved [60]. There were no serious side effects from levetiracetam in the study but 2 children experienced mild side effects, one with a mild increase in appetite and another with an increase in daytime irritability [60]. Prescribers should be aware of the possibility of the development of rare but severe cutaneous reactions such as the Stevens-Johnson syndrome. Furthermore, treatment guidelines also contain a warning for use in renal failure. Psychiatric side effects, including psychosis, paranoid ideation, and behavioral instability, may also occur [61].

### 3.5. Steroids And The Auto-Immune, Anti-Inflammatory Link In Restless Legs Syndrome

RLS can be triggered de novo by infections and RLS is also characterized by the elevation of inflammatory and hypoxic markers [62]. In addition, RLS occurs more commonly than one would expect by chance alone in association with disorders that have an auto-immune diathesis such as Multiple Sclerosis, Rheumatoid Arthritis, and Celiac Disease [62]. Given these associations, it is not surprising that RLS could be modulated by anti-inflammatory agents such as steroids [63,64]. In a double-blind crossover trial, 10 patients received 40 mg of hydrocortisone or saline as a placebo prior to a one-hour SIT [63]. Sensory leg discomfort as measured on a visual analogue scale was significantly lower with hydrocortisone than with placebo (p =0.032) [63]. Five of 10 patients experienced subjective improvement of RLS symptoms with hydrocortisone during the SIT (mean visual analogue scale of sensory leg discomfort was 70 with placebo vs. 40 in the hydrocortisone group; p < 0.05) but this was true of none of the patients during the placebo portion of the study [63]. None of the patients had side effects in this very short-term study [63]. In a case report of a single RLS patient, oral prednisolone was administered first at a dosage of 15 mg/day [64]. After the first 4 weeks of therapy, his IRLS scale score fell from 22 to 14, his PLMS index fell from 24 to zero, and his ESS dropped from 14 to 5 (no p-value provided) [64]. The prednisolone was then tapered to a minimum effective dose of 8 mg/day and the patient remained well on this dosage for 12 months at the time of publication [64]. Side effects were not given to the reader in this single case report [64]. If steroids are administered long-term, patients would need to be monitored carefully for the development of long-term consequences such as osteoporotic fractures, hypertension, steroid-induced diabetes, and changes in physical appearance such as moon face and buffalo hump [64,65,66].

### 3.6. Other Medications And Substances

#### 3.6.1. Cannabinoids

The positive response to cannabinoids in the following two case reports [67,68] suggests that the cannabinoid receptor may be downregulated in RLS, although we know of no basic science inquiries into this possibility at present. In the first of the case reports, 6 patients from France with long duration (5–23 years) and very severe RLS (IRLS scale score range from 32 to 37 on a 40-point scale) reported that they on their own had taken cannabis for their RLS with complete resolution of symptoms (no p-value provided) despite having previously failed multiple standard RLS medications such as dopamine agonists, gabapentin, pregabalin, and opioids [67]. Cannabis in this study was reported to be well tolerated compared to previous therapies [67]. The second study was a survey in Canada on RLS and pruritis in patients with end-stage kidney disease on dialysis. The study indicated that of the 192 patients who returned the survey 86 (45%) had RLS [68]. Six of these 86 were currently asymptomatic on treatment [68]. Fifteen had tried cannabis for their RLS symptoms and 9 had noted improvement (no score or p-value provided) [68]. Fifty-six of the 81 symptomatic RLS patients (70%) were interested in a future trial of cannabis in RLS [68]. Patients were not surveyed for the presence or absence of side effects in this study [68]. The ability to use Cannabis for RLS varies from country to country and from state to state within the United States. Cannabidiol (CBD) has less psychoactive active effects, is generally more legally available, and could theoretically be useful for RLS as well. The effect of different types of cannabis or their derivatives may influence symptoms differently. Prescriber guidelines include monitoring for potential CNS depression, hepatic impairment, and suicidal ideation [69].

#### 3.6.2. Bupropion

Bupropion is often chosen as an antidepressant for RLS patients with concomitant depression as it has pro-dopaminergic properties and does not exacerbate RLS [70]. There is some evidence from the literature that bupropion may also have an ameliorating effect upon RLS symptoms as documented in two case reports, each of a single patient [71,72] and in another case series of 3 patients [73] (no p-value provided in either study). The dosages in the studies ranged from 150–300 mg XL administered once daily [71,72,73]. Interestingly morning administration was effective for night-time symptoms of RLS in the 3-patient case report [73]. Two larger studies also explored the effect of bupropion on RLS [74,75]. In the first, 29 patients received bupropion 150 mg XL/day and 31 patients received a placebo for 6 weeks in a double-blind study [74]. At 3 weeks there was a statistically significant improvement in the IRLS scale score (mean –10.8 points from baseline in bupropion vs. –6 points with placebo; p = 0.016) but this improvement did not quite reach statistical significance at 6 weeks (mean –10.4 points from baseline in bupropion vs. –7.6 points with placebo; p = 0.108) [74]. Two patients in the bupropion group withdrew from the study, one due to nausea and the other due to a miscarriage [74]. In the second double-blind study, 90 patients were divided into groups of 30 each with each group receiving either bupropion 300 mg/day, ropinirole 0.5 mg/day, or oral iron 150 mg elemental formulation along with vitamin C [75]. All 3 groups showed statistically significant improvement in mean improvement in IRLS scale score severity (Bupropion: –6 points; Ropinirole: –15 points; Iron: –3 points; p = 0.01) and RLS Quality of Life (p = 0.002), but for the IRLS scale score the ropinirole group improved more than bupropion or iron (Bupropion: –5 points; Ropinirole: –22 points; Iron: –4 points; p = 0.001) [75]. Only one patient in the bupropion group had a side effect and this was mild gastritis which did not result in study drop-out [75]. Treatment guidelines contain a boxed warning alerting prescribers to the possibility of suicidal thoughts and behavior [76].

#### 3.6.3. Baclofen

Baclofen is a muscle relaxant that was originally noted to improve PLMS in 5 patients [77]. However, the results for baclofen therapy in RLS are limited and conflicting as one patient improved with baclofen 10 mg nighty at sleep [78] and one with intrathecal baclofen did not [79] (no score or p-value for either study). Side effects from baclofen were not reported in either of the cases [78,79]. Treatment guidelines recommend withdrawing patients on maintenance intrathecal baclofen very slowly because of the possibility of developing a high fever, confusion, and, in some cases, rhabdomyolysis, multiple organ failure and death [80].

#### 3.6.4. Physostigmine

Physostigmine is an anticholinesterase inhibitor. Under the right circumstances, it can be administered intra-operatively or intra-procedurally. Two separate case reports of one RLS patient each demonstrated relief with the injection of 1 mg intravenous (iv) of physostigmine (no score or p-value provided) [81,82]. Side effects were not reported after the single injection of physostigmine in either patient [81,82]. The short half-life of this drug and the intravenous mode of administration may limit its applicability as a more practical and widely used therapy [83]. Other treatment guideline contraindications of using physostigmine in patients with gastrointestinal or genitourinary obstruction, asthma, cardiovascular disease, and may cause arrhythmias, or seizures [83].

#### 3.6.5. Orphenadrine citrate

Orphenadrine citrate is a muscle relaxant. In a study of 32 patients, where there was not a clearly separated mixture of patients with RLS and leg cramps, orphenadrine citrate 100 mg–200 mg/day in 1 or 2 divided doses in the evening was administered to the subjects for from 3 months to 1 year [18]. Twelve subjects were discontinued from the study because they had gastrointestinal intolerance to the medication, took the medication only sporadically, or were not reliable observers [18]. Sixteen of the remaining 20 subjects experienced long-term excellent results based upon subjective reports (no score or p-value provided) [18]. Treatment guidelines suggest that orphenadrine citrate not be used in older adults because of an increased risk of anticholinergic effects, sedation, and risk of fracture. Prescribers are also warned to use with caution in patients with heart disease and drug or alcohol abuse [84].

#### 3.6.6. Rifaximin

An association of RLS to small intestinal bacterial overgrowth (SIBO) has been established [85,86]. SIBO may lead to RLS since inflammation may lead to iron deficiency, a known precipitant of RLS [85]. Another possibility is that, as a result of SIBO, there is a direct auto-immune attack upon the central or peripheral nervous system leading to RLS [62]. In support of this hypothesis is the observation that many of the co-morbid conditions associated with RLS have an autoimmune diathesis [62]. Rifaximin is an antibiotic that improves SIBO symptoms when used in a titrated schedule of 400mg three times a day for 10 days with tapering to 400 mg every other day for the next 20 days [86]. A small clinical study that enrolled patients with Irritable Bowel Syndrome (IBS) found that there were 13 patients who met the criteria for RLS [86]. These 13 individuals were assigned into a group that took rifaximin 400 mg three times daily for 10 days followed by a daily probiotic (n = 11) or rifaximin monotherapy of 400 mg three times daily for 10 days (n = 2) [86]. At the end of the study period, nearly half (6/13) of patients had complete resolution of RLS symptoms, and 86% (12/13) of patients had an overall improvement in RLS symptoms compared to baseline in long-term follow-up of a mean of 139 days, with 46% of patients (6/13) experiencing complete resolution of RLS symptoms (no score or p-value provided) [86]. There was also a significant improvement in IBS symptoms including abdominal distention, diarrhea, and bloating in over two-thirds of the patients [86]. However, a validated scale like the IRLS was not used, and instead, these findings were based on a categorial variable measured in a post-intervention questionnaire that used arbitrary categories of 4-point Likert scale (greatly, moderately, mildly, or no improvement) in RLS symptoms [86].

In the second open-label study by the same authors, the IRLS was employed as an endpoint [87]. Twenty-one patients with RLS were screened for SIBO which was found in 14 who were treated with Rifaximin 1200 mg/day for 10 days followed by 400 mg every other day for 20 days [87]. Eleven of the subjects had other concomitant GI diseases such as irritable bowel syndrome, functional pain/bloating, Crohn’s disease, and Celiac disease [87]. At baseline, IRLS scale scores were 23.1 on average [87]. Nine of the 14 patients reported global improvement in RLS symptoms and were designated responders (no score or p-value provided) [87]. The mean percentage improvement in IRLS scale scores was 65.6% in responders versus – 5.7% in non-responders (no p-value provided) [87]. These early studies suggest that SIBO associated with IBS is an important consideration to keep in mind when managing patients with RLS who suffer from gastrointestinal symptoms. Side effects of the use of rifaxamin were not reported by the authors in either study [86,87]. Treatment guidelines for rifaximin use include warnings to observe for allergic reactions and super-infections with other organisms [88].

#### 3.6.7. Botulinum toxin (BTX)

For Botox and RLS or Botulinum toxin and RLS our search yielded 7 publications. The use of botulinum toxin (BTX-A; 100 units per cc) in RLS ranged from 25–320 units administered to different sites for the treatment of refractory RLS. [89]. One of the 3 subjects experienced residual weakness [89]. A randomized double-blind placebo-controlled crossover study using incobotulinum toxin (100 units IncoA) or saline injected in the tibialis anterior, gastrocnemius, and biceps femoris bilaterally, improved the severity of symptoms from severe to mild-moderate at 6 weeks in 24 patients using the IRLS scale (p = 0.033). None of the subjects were reported to have residual weakness [90]. An open-label case series of 8 RLS subjects receiving BTX on the anterior tibialis, demonstrated improvement in the IRLS (–5.8 points; p = 0.029) during the first four weeks after injection (50 units of onabotulinum toxin A). None of the subjects were reported to have residual weakness [91]. In another open-label study using BTX A in various anterior and posterior leg areas of 27 patients, 6 patients had improved symptoms by >50% in the IRLS scale at week 2 from baseline (p < 0.001). Seven of the 27 subjects were reported to have transient limb weakness and 1 was reported to have diplopia [92]. Lastly, in a double-blind, placebo-controlled study, BTX A (20 intradermal injections of 0.05 ml of BoNT/A) or saline was injected in quadriceps femoris, tibialis anterior, gastrocnemius, and soleus of 6 patients without showing significant improvement at week 2 in the IRLS (+1 point improvement in BTX vs. +5 point improvement with placebo; p = 0.06) or CGI scales (+2.8 point improvement with placebo vs. +4.3 point improvement with placebo; p = 0.01). Two subjects experienced weakness [93]. Prescriber guidelines include a black box warning of dysphagia and breathing difficulties, bacteriuria, urinary retention and tract infection, and anaphylaxis hypersensitivity reaction [94].

In summary, there is no standard protocol for the administration of BTX in patients with RLS. Dosage varies among studies from 50 U to 100 U per leg to 500–1000 U per patient. Current studies in a small number of patients have failed to produce robust evidence in favor of the use of BTX in RLS which may be influenced by the limited number of patients, the dose, or the technique used. Furthermore, case reports or studies without controls make it harder to rule out placebo effects. Patients must be observed for temporary excess weakness [95].

#### 3.6.8. Selegiline

Although selegiline increases dopamine levels, it does it by a different mechanism than dopamine receptor agonism. It has not been studied well enough to be considered a mainstay of therapy for RLS or PLMS. Selegiline is a monoamine oxidase (MAO) inhibitor type B and it also interferes with dopamine reuptake at the synapse. In an open-label study of selegiline in 31 patients with PLMS where RLS was excluded, patients were slowly titrated from 5 mg to 10mg to finally 15 mg of selegiline bid in two-week intervals [96]. PLMS were suppressed in the total group by 21.2/hr (pretreatment 35.6/hr and post-treatment 14.5/hr) (P < .0005) as determined by polysomnography before and after treatment [96]. The subgroup of 21 patients who were on 15mg bid treatment also improved by a reduction of PLMS of 20.7/hr (p < .0005) [96]. Four subjects experienced slight feelings of disconnection, nervousness, and nausea [96]. However, these side effects did not lead to discontinuation either before or after the second sleep study [96]. Undesired alerting effects at bedtime were not statistically significant [96]. Prescribing guidelines for using selegiline include orthostatic hypotension, vertigo, palpitation, suicidal thinking/behavior, dyskinesia, psychosis, and CNS depression [97].

## 4. Discussion and Future Directions for Pharmacological Treatment of RLS

### 4.1. Serotonin

It is well known that selective serotonin re-uptake inhibitors such as paroxetine which increase the synaptic concentration of serotonin also worsen RLS [29,46,70]. This would suggest that RLS is characterized by a hyper-serotonergic state. This was confirmed in a study that showed that the availability of serotonin transporters on neuroimaging in patients with RLS decreased as the severity of RLS increased [98]. This suggests that medications that could lower serotonin levels could be a useful therapy for RLS. An example of a serotonin antagonist is cyproheptadine, used to commonly treat excess serotonin, although providers should be aware that no studies exist on its effect on RLS and that the medication has strong CNS depressive and anticholinergic properties that should be avoided in patients >65 years of age [99].

### 4.2. Histamine

RLS is also worsened by first-generation H1 receptor-blocking antihistamines [29]. In addition, basic science studies indicate that striatal H3 receptor levels positively correlate with the number of PLMS in an iron-deficient rodent model of RLS. Blocking the H3 receptor lowered the number of PLMS [100]. The authors suggest that H3 receptor antagonists could be a potential therapy for RLS in humans [100]. In this light, it would be interesting to see the effects of pitolisant on RLS as it is an H3 receptor antagonist/reverse agonist recently approved in the United States for the treatment of narcolepsy and cataplexy [101]. However, the general relationship between RLS and narcolepsy may be more remote. Orexin/hypocretin deficiency is the main cause of human narcolepsy but variations of the levels and circadian timing of hypocretin-1 have been measured in the CSF of RLS patients with inconsistent results [102,103,104]. Pitolisant prescribing guidelines include that it can cause hepatic impairment, QT prolongation, and headache [105].

### 4.3. GABA

Eighteen patients with RLS and a matched control group were studied with proton magnetic resonance spectroscopy and actigraphy [106]. Levels of gamma-aminobutyric acid (GABA) did not differ between RLS patients and controls [106]. However, GABA levels correlated positively with the PLMS index and RLS severity in the thalamus and negatively with these parameters in the cerebellum [106]. In another study, GABA receptor gene polymorphisms were studied in 205 RLS patients and 230 age and sex-matched controls [107]. The GABRR3 (rs832032) T allele was significantly correlated with RLS and the GABRA4 (rs2229940)TT genotype was associated with younger age of onset of RLS [107]. From the aforementioned studies, it is difficult to know whether any future therapy should be aimed at increasing or decreasing GABA levels. However, a therapeutic trial with a medication whose primary mode of action is GABA-mediated might be warranted. Such a drug could be the anticonvulsant tiagabine whose primary mode of action is to enhance the action of GABA through transporter inhibition [108]. To our knowledge, no studies have been done on GABA or tiagabine in RLS.

### 4.4. Limitations

There are some limitations to our study. We have performed a scoping review based on one search engine, PubMed. We did not apply any grading of evidence, because those studies with high level of evidence and that are standard care of treatment were not considered for our review per our exclusion criteria. Therefore, the remaining articles which we reviewed are potentially all of a lower level of evidence.

### 4.5. Conclusion

We have reviewed medications that are lesser known as treatments for RLS. This is not an evidence-based review because the supportive data for these lesser proven, and thus lesser known treatments is, by definition, low. We would emphasize that clinicians should first turn to more well-proven treatments for RLS including the alpha delta calcium channel anticonvulsants gabapentin and pregabalin, the dopamine agonists pramipexole, ropinirole and rotigotine, the opioids in refractory cases and to oral and intravenous iron [1,2,3,4,5,6,7,8]. However, many times in clinical practice the more well-proven agents produce intolerable side effects or are incompletely effective. In such cases, clinicians should be aware of additional therapeutic options. We neither recommend nor not recommend such options but leave it up to the clinician to make their own decision based upon the therapeutic and side effect profile of each agent. Being aware of such options is the first step toward potentially better patient care.

**Figure:**
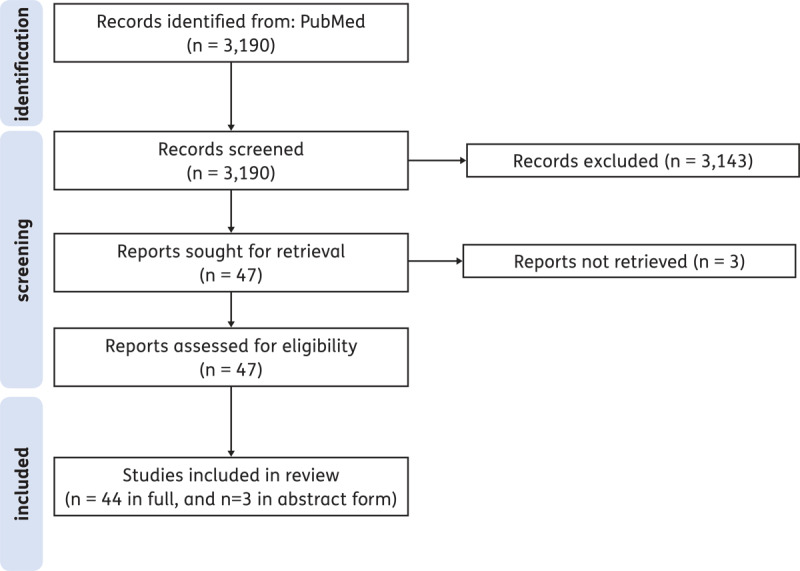
Supplementary Figure S1 Flowchart of the selected studies.

Among the agents, that we have reviewed are clonidine which causes a reduction in adrenergic transmission, adenosinergic agents such as dipyridamole, glutamate AMPA receptor blocking agents such as perampanel, glutamate NMDA receptor blocking agents such as amantadine and ketamine, various anticonvulsants (carbamazepine/oxcarbazepine, lamotrigine, topiramate, valproic acid, levetiracetam), anti-inflammatory agents such as steroids, as well as cannabis. Bupropion is a good choice for the treatment of co-existent depression in RLS because of its pro-dopaminergic properties. We would emphasize that we have only reviewed medications and not non-pharmacologic, herbal preparations, vitamins, minerals, compression, electrical stimulation, or vibration devices that have been reviewed elsewhere and may provide additional therapeutic options [15,16].
